# Healthcare-associated fungal infections and emerging pathogens during the COVID-19 pandemic

**DOI:** 10.3389/ffunb.2024.1339911

**Published:** 2024-02-23

**Authors:** Krish Shah, Mukund Deshpande, P. Shah

**Affiliations:** ^1^ Biological Sciences Bellarmine College Preparatory, San Jose, CA, United States; ^2^ R&D Center, Greenvention Biotech Pvt. Ltd., Pune, India; ^3^ Science Ambassador/Bio-Rad Laboratories, Hercules, CA, United States

**Keywords:** antifungal drugs, COVID-19, emerging fungal pathogens, hospital infections, resistance

## Abstract

Historically, fungi were mainly identified as plant and insect pathogens since they grow at 28°C. At the same time, bacteria are known to be the most common human pathogens as they are compatible with the host body temperature of 37°C. Because of immunocompromised hosts, cancer therapy, and malnutrition, fungi are rapidly gaining attention as human pathogens. Over 150 million people have severe fungal infections, which lead to approximately more than one million deaths per year. Moreover, diseases like cancer involving long-term therapy and prophylactic use of antifungal drugs in high-risk patients have increased the emergence of drug-resistant fungi, including highly virulent strains such as *Candida auris*. This clinical spectrum of fungal diseases ranges from superficial mucocutaneous lesions to more severe and life-threatening infections. This review article summarizes the effect of hospital environments, especially during the COVID-19 pandemic, on fungal infections and emerging pathogens. The review also provides insights into the various antifungal drugs and their existing challenges, thereby driving the need to search for novel antifungal agents.

## Introduction

Fungi possess one of the following characteristics as human pathogen**s**. Certain fungi, for instance, have the ability to change their vegetative morphology. To survive and proliferate in human hosts, they can exist as a filament or as a unicellular form (Ghormade and Deshpande, 2000). Some have a high degree of melaninization, which does not allow antifungal drugs to enter the cell very easily (Butler et al., 2001). Sometimes, the capability to grow under extreme conditions also makes certain fungi human pathogens. Fungal infections in humans occur when fungal pathogens invade and colonize host tissues, resulting in various disease manifestations. The exact mechanisms by which fungal pathogens cause disease in humans can vary depending on the specific pathogen, the host’s immune response, and numerous other factors. In general, the steps for pathogenesis include adhesion, invasion, immune evasion, host tissue damage, and fungal persistence. Adhesion usually involves the binding of fungal cell surface proteins to host cell receptors. This correlation has been established by the construction of deletion mutants of pathogens and the demonstration of their reduced adherence and virulence. The most known fungal adhesins are glycosylphosphatidylinositol (GPI)-modified cell wall proteins (de Groot et al., 2013). *Aspergillus fumigatus* causes aspergillosis in patients who have AIDS, solid-organ transplants, or chronic pulmonary diseases and in patients with various hematological malignancies. Adhesin, or RodA, is one of the most extensively studied proteins for its interaction with host cells. Invasion provides a mechanism for the pathogen to traverse natural cellular barriers such as the vascular endothelium or pulmonary epithelium. Hydrolytic enzymes, such as proteinases, lipases, and phospholipases, help the organism invade host tissue (Park et al., 2011). Moreover, invasion into endothelial or epithelial cells protects the fungus from phagocytes. In addition, the host cell also serves as a reservoir of nutrients for the fungus (Sheppard and Filler, 2015). Fungal pathogens have developed various mechanisms to evade or suppress host immune responses, which include the secretion of proteases, toxins, and superantigens. *Cryptococcus neoformans* was not usually considered a cytotoxic fungal pathogen until it was extensively studied to demonstrate its ability to damage host cells (Casadevall et al., 2018). This host cell damage occurs due to lytic exocytosis, organelle dysfunction, phagolysosomal membrane damage, and cytoskeletal alterations. Fungal pathogens such as *C. neoformans, Candida glabrata*, and *Histoplasma capsulatum* can manipulate host immunity even after phagocytic uptake by blocking phagolysosomal maturation or acidification (Brown, 2022). The World Health Organization (WHO) published a fungal priority pathogen list consisting of 19 pathogens (**Table 1**), divided into three key groups (Parums, 2022).

**Table 1:** Priority fungal pathogens adapted from The World Health Organization Fungal Pathogen Priority List, 2022.

Pathogen Category	Fungal Species Involved
Critical priority	*Aspergillus fumigatus*, *Candida albicans, Candida auris*, and *Cryptococcus neoformans*
High priority	*Candida glabrata, Candida parapsilosis*, *Candida tropicalis*, along With *Fusarium* species, *Histoplasma* species, *Mucorales* species and mycetoma causing fungi
Medium priority	*Candida krusei, Coccidiodes* species, *Cryptococcus gattii, Lomentospora prolificans, Scedosporium* species, *Paracoccidiodes* species, *Pnuemocystis jirovecii*, and *Talaromyces marneffei.*

## Emerging fungal pathogens

Emerging fungal pathogens change the landscape of human mycology (Friedman and Schwartz, 2019). This can be attributed to autoimmune diseases, which have added new risk factors. For instance, non-albicans *Candida* species increasingly cause invasive candidiasis. Similarly, non-fumigatous *Aspergillus*, mucoraceous fungi, and *Scedosporium* infections have increased due to antifungal prophylaxis in cancer patients. New emerging pathogens like azole-resistant *A. fumigatus, Emergomyces, Trichophyton, Blastomyces*, and *Sporothrix* are increasing all over the world. The new emerging pathogens are usually non-pathogenic relatives of existing established pathogens. Sometimes, emerging pathogens are resistant to conventional antifungal therapy and may cause severe morbidity and mortality in immunocompromised patients. Some examples of emerging pathogens include *Acremonium, Bipolaris, Fusarium, Paecilomyces, Scedosporium, Trichoderma, Trichosporon*, and several zygomyceotus species. In a recent study, Kim et al. (2020) extensively reviewed the human healthcare problems caused by plant-pathogenic fungi, bacteria, and viruses. Plant pathogens like *Alternaria, Cladosporium*, and *Colletotrichum* species, which cause leaf spot and blight in their respective plant hosts, have also been reported to cause blood, ophthalmic, and respiratory tract infections in humans. *Ustilago maydis*, which causes corn smut, is known to cause skin lesions (Teo and Tay, 2006). *Conidiobolus coronatus*, an insect pathogen, can cause nasal polyps and sinus infections in people (Vilela and Mendoza, 2018). Additionally, *Schizophyllum commune*, a wood-decomposing fungus, has been found in human sputum, is known to grow on human fingers and toenails, and causes sinusitis (Premamalini et al., 2011). *S. commune* also leads to fungal ball formation in the lung cancer cavity (Itoh et al., 2021). To summarize, **Table 2** illustrates emerging fungal pathogens that were originally identified as plant or insect pathogens.

**Table 2:** Emerging fungal pathogens.

Fungal Species	Primary Host	Emergence as human pathogen	References
*Alternaria, Cladosporium*, *Colletotrichum* species	Plants/ Leaf spot/Blight	Ophthalmic and respiratory tract infections in humans	Kim et al., 2020
*Ustilago maydis*	Corn smut	Skin lesions	Teo and Tay, 2006
*Conidiobolus coronatus*	Insect Pathogen	Nasal polyps/Sinus infections	Vilela and Mendoza, 2018
*Schizophyllum commune*	Wood-decomposing fungus	Grows on human fingers and toenails and causes sinusitis, fungal ball formation in lung cancer	Premamalini et al., 2011; Itoh et al., 2021

## Healthcare-associated fungal infections and the Covid-19 pandemic

Fungal pathogens are detrimental to human health and can cause a range of adverse health repercussions, ranging from mild skin infections to life-threatening systemic infections. Within a hospital setting, fungal pathogens can enter the human body through cuts, wounds, burns, surgical sites, and inhalation. In ICU patients, *Candida* and *Aspergillus* coinfections were frequently observed in different settings. It was observed that COVID-19 increased the risk for invasive fungal infections (IFIs) because of its treatments like steroids and other drugs, which negatively affected the patient’s defense mechanism against fungi. Symptoms of certain fungal diseases are like those of COVID-19, including fever, cough, and shortness of breath. Additionally, some patients can have COVID-19 and a fungal infection at the same time. The most reported fungal pathogens were *Aspergillus, Candida, Mucor*, and other mucoralean genera. Fungal infection becomes serious because of the delay in its detection and treatment. A recent review highlighting fungal coinfections in COVID-19 patients claimed that more than 50% of the patients suffering from COVID-19 contracted secondary infections, resulting in death. There are reports that fungal coinfections increased the death rate in COVID-19 patients (Seyedjavadi et al., 2022). **Table 3** summarizes the rate and type of fungal coinfections in COVID-19 patients admitted to the ICU across various continents. For instance, 72.9% of the patients with co-infections were male and 25.9% were female. Patients in the age group of less than 50 years had a 23.7% chance of coinfection, while patients greater than 50 years of age had a 66.2% chance of fungal coinfection. Therefore, COVID-19 increases the chance of acquiring fungal infections. The disease pattern of COVID-19 can range from mild to severe pneumonia associated with bacterial or fungal infections (Mehta and Pandey, 2020). Due to related complications such as hypertension, diabetes, and immunocompromised conditions, COVID-19 patients are very likely to succumb to fungal infections. In India, COVID-19-associated mucormycosis was observed to be a significant public health problem, leading to significant health-related concerns. Uncontrolled diabetes and overuse of steroids for COVID-19 treatment were significant risk factors for its growth (Abdalla et al., 2020). Usually, *Mucor* species change their morphology from mycelium to unicellular yeast in the presence of high blood glucose (Ghormade et al., 2011). In March 2023, there was an alert from the Center of Disease Control and Prevention that in hospitals, fungus *C. auris* was spreading rapidly because of its resistance to various antifungal agents (Lyman et al., 2023). *C. auris* infections were first reported in Japan in 2009, and since then, they have spread in different countries. The infection of *C. auris* spreads through wounds, the urinary tract, and the bloodstream. In hospitals, it stays on various plastic and other surfaces for up to fourteen days and forms biofilms. It also produces tissue-damaging hydrolytic enzymes like phospholipases and proteinases. The possible reasons for the increase in new fungal infections were the increasing number of people getting sick and hospitalized, concurrent with the pressure on the health system. As a result, during the COVID-19 pandemic, fungal pathogens increased the chance to induce life-threatening diseases (Gold et al., 2023). The irony of the situation is that medical advances save patients, but at the same time, they put individuals, especially those on monoclonal antibody-based treatments for various auto-immune diseases, at risk of catching vulnerable fungal infections. For example, steroids are known to be immune-suppressive by impairing T lymphocyte activation. They do this by blocking the expansion of the T helper cells. Early use of steroids affects immune cells and impairs immunotherapy efficacy in the treatment of patients with non-small cell lung cancer (NSCLC) (Della Corte and Morgillo, 2019). During the COVID-19 pandemic, fungal pathogens such as *Aspergillus, Candida*, and *Mucor* were commonly found in hospital patients. *C. auris* infection was observed in acute care hospitals due to extended use of gloves, gowns, and changes in disinfection practices. One of the primary reasons for this was the development of resistance in the pathogens to the existing antifungal drugs. Moreover, rapid, and sensitive detection methods for the pathogens are not available. Earlier, outbreaks of primary cutaneous aspergillosis and central nervous system aspergillosis were associated with using contaminated medical devices (Allo et al., 1987; Vonberg and Gastmeier, 2006). The prevalence of COVID-19 increases the risk for severe fungal infections because of the effects on related immune system function, structural lung damage, and treatments (e.g., corticosteroids and immunomodulatory drugs) that impair host defenses against fungal pathogens (Holshue et al., 2020). Compared with non-COVID-19-associated deaths, COVID-19-associated deaths more frequently involved *Candida* (27.1%), *Aspergillus* (23.3%), and less frequently involved other specific fungal pathogens (Baddley et al., 2021). Another example is related to mucormycosis; when the COVID-19 wave ravaged India, it left several recovering patients with compromised immune systems. The pathogen colonizes blood vessels, and thus, infection is angio-invasive, resulting in thrombosis and tissue necrosis. Aspergillosis and mucormycosis infection rates were higher in patients with diabetes, who were also immunocompromised due to the severe COVID-19 infection, followed by steroid treatment (Abdalla et al., 2020).

**Table 3:** Rate and type of fungal coinfections in Covid-19 patients admitted to ICU across various continents (Seyedjavadi et al., 2022).

Continent	% of ICU admitted patients with fungal coinfections	Reported Fungal Coinfection
America	23.2	Invasive Aspergillosis, Mucormycosis, Candidiasis and Cryptococcosis
Asia	49.7	Invasive Aspergillosis, Mucormycosis, Candidiasis and Cryptococcosis
Europe	19.8	Invasive Aspergillosis, Candidiasis
Australia	0.55	Invasive Aspergillosis
Africa	6.6	Invasive Aspergillosis, Candidiasis

## Antifungal drugs and existing challenges

Currently, several classes of drugs are used to treat invasive fungal infections (Wall and Lopez-Ribot, 2020). The azoles inhibit ergosterol synthesis, resulting in the accumulation of toxic intermediates and loss of membrane integrity (Whaley et al., 2016). They have broad-spectrum activity. However, the major constraints include the development of resistance to pathogens, low oral bioavailability, and hepatic toxicity. Furthermore, Amphotericin B and Nystatin are two polyene antibiotics. Amphotericin B is fungicidal since it binds to ergosterol (Gray et al., 2012). It extracts sterols from the cell membrane of fungi, leading to a weakened cell membrane. As a result of this, there is leakage of cytosolic contents that ultimately results in cell death. However, renal dysfunction is a main negative point for its use since it is nephrotoxic. Similarly, nystatin is also restricted to topical application due to host toxicity. Echinocandins are the most recent class of FDA-approved antifungal agents and are known to be glucan synthesis inhibitors, ultimately leading to the disruption of the fungal cell wall. Caspofungin was the first approved echinocandin for use in humans in 2001 (Sucher et al., 2009). Since mammalian cells do not have a cell wall, echinocandins are considered the safest antifungal agents. Allylamines inhibit ergosterol synthesis like azoles, but the target enzymes are squalene epoxidase and lanosterol 14-α-demethylase. Terbinafine, for example, is fungicidal against many fungi because it inhibits squalene epoxidase. When fungi accumulate squalene, their ergosterol levels go down (Ryder, 1992). Ergosterol is an essential element of the fungal cell membrane. Fluorinated pyrimidine compounds like 5-fluorocytosine, which affect DNA synthesis, have limited use because of their hematological toxicity, rapid development of resistance, and effect on non-target cells. The status of fungal infections and the drawbacks of existing drugs, including acute and chronic side effects, less clinical efficiency, and effects on non-targeted cells, demand the development of more effective, safe antifungal agents with novel targets. It is indeed evident from the various reports that the rate of fungal infections increased during the COVID-19 pandemic, particularly in patients who have been hospitalized or received treatment in intensive care units. This increase in fungal infections can be attributed to broad-spectrum antibiotics, corticosteroids, and other medications that can weaken the immune system. COVID-19 patients who receive treatment in hospitals or ICUs may be at increased risk for this infection due to the use of immunosuppressive medications and the prolonged use of oxygen therapy. Furthermore, the COVID-19 pandemic has led to disruptions in the global supply chain for antifungal medications (Bienvenu et al., 2022), making it more difficult for healthcare providers to treat fungal infections. This shortage is particularly problematic in developing countries, where access to antifungal medications is already limited. Overall, the COVID-19 pandemic has created conditions that may increase the risk of fungal infections and exacerbate existing challenges in managing these infections.

## Model organisms to screen and develop antifungal agents

Pathogenic fungi such as *C. albicans* are polymorphic and undergo morphological changes. They adopt forms like unicellular yeast or filamentous mycelium to ensure their survival and proliferation within a host and overcome the host’s cellular defenses. They have two glutamate dehydrogenase enzymes that potentially play an important role in *C. albicans* morphogenesis when the fungus switches between yeast and filamentous forms (Rooney and Klein, 2002). The biochemical processes associated with this transition from a less virulent to a pathogenic state can be potential targets for developing innovative antifungal agents. Under favorable conditions, both asexual and sexual spores germinate into forms like unicellular yeast or filamentous mycelium (Pathan et al., 2017), facilitating invasion into susceptible hosts.

For instance, *H. capsulatum’s* sexual ascospores can germinate into yeast-like cells at 37°C, indicating their potential for host invasion. Similarly, in the case of *Cokeromyces recurvatus*, a dimorphic zygomycete, sporangiospores have been observed to germinate into unicellular yeast, serving as infective propagules for pathogenesis. A non-pathogenic zygomycetous dimorphic fungus, *Benjaminiella poitrasii*, exhibits temperature-induced dimorphism like pathogenic fungi such as *H*. *capsulatum* and *P*. *brasiliensis*. Therefore, it is valuable to develop antifungal agents that can target biochemical events triggering the yeast-hypha transition (Ghormade and Deshpande, 2000; Pathan et al., 2017). The enzymes involved in cell wall synthesis and degradation will be potential targets for antifungal drugs. For instance, pre-clinical studies for nikkomycin, a chitin synthesis inhibitor, are in progress (Chaudhary et al., 2013; Pathan et al., 2017). Pathan et al. (2019) have shown a cause-and-effect relationship between glutamate dehydrogenase enzymes and yeast-hypha transition, the morphological outcome in *B. poitrasii.* The ability of a pathogen to switch between yeast and filamentous growth is one of the virulence attributes of human pathogenic fungi. In other words, inhibiting glutamate dehydrogenase activity can hamper morphological transition for survival and eventually control pathogenesis caused by fungi such as *C. albicans* (Han et al., 2019).

## Epilogue

The main concern for fungal infections is their increased risk in a hospital environment, especially during a pandemic. In most cases, the infections are more common in the patients under treatment in ICUs. The irony of the situation is that medical advances save patients, but at the same time, they put individuals at risk of catching vulnerable fungal infections. Given the current challenges associated with fungal infections, including limitations of existing drugs such as acute and chronic side effects, suboptimal clinical efficiency, and impacts on non-targeted cells, there is a pressing need to develop more effective and safe antifungal agents. Novel targets like chitin synthase, specific to fungi and absent in other eukaryotic cells, present promising avenues for designing antifungal drugs with enhanced specificity and reduced adverse effects. The fungal cell membrane already exists as a promising drug target for antifungal therapy (Sant et al., 2016). Other targets, like ornithine decarboxylase glutamate dehydrogenase, shared by other eukaryotes, have different structures in fungi and can offer promising drug targets for antifungal screening.

## Author contributions

KS: Conceptualization, Data curation, Writing – original draft, Writing – review & editing. MD: Conceptualization, Data curation, Supervision, Writing – original draft, Writing – review & editing. PS: Data curation, Supervision, Writing – review & editing.
